# Differential Performance of Vector and Non-Vector Planthoppers on Virus-Infected vs. Mock-Infected Plants

**DOI:** 10.3390/insects17060631

**Published:** 2026-06-15

**Authors:** Guangchao Cui, Pei Li, Somkhit Sengsay, Artisack Seesomphone, Laythong Sisongkham, Kongkham Akhavongsa, Huai Liu, Maolin Hou

**Affiliations:** 1 State Key Laboratory for Biology of Plant Diseases and Insect Pests, Institute of Plant Protection, Chinese Academy of Agricultural Sciences, Beijing 100193, China; 821012490031@caas.cn (G.C.);; 2 College of Plant Protection, Southwest University, Chongqing 400715, China; liuhuai@swu.edu.cn; 3 Plant Protection Center, Department of Agriculture, Ministry of Agriculture and Environment, Vientiane P.O. Box 811, Laos

**Keywords:** southern rice black-streaked dwarf virus, *Sogatella furcifera*, *Nilaparvata lugens*, performance, salicylic acid, gene expression

## Abstract

The southern rice black-streaked dwarf virus (SRBSDV) is transmitted by the white-backed planthopper (WBPH), *Sogatella furcifera*, but not by the co-occurring brown planthopper (BPH), *Nilaparvata lugens*. Understanding the influence of virus infection of host plants on the performance of close-related vector and non-vector species is an interesting topic for exploring virus–plant–herbivore interactions. This study investigates how SRBSDV infection of rice plants affects the performance of WBPH and BPH and the plant defense responses. Differential performance of the two planthopper species was observed. On infected plants, WBPH displayed prolonged male nymphal development, increased adult longevity, enhanced feeding, and reduced fecundity, which contrasts the reduced nymph survival and fecundity in BPH. SRBSDV infection triggered an increase in salicylic acid (SA) levels and upregulated the expression of SA-related genes (*ICS1* and *NPR1*) in response to WBPH feeding, but not to BPH feeding. These results show that SRBSDV reshapes the host plant defense in a manner that alters key vector traits favoring virus transmission while impairing the fitness of a competing non-vector, which advances current understanding of virus–plant–herbivore interaction.

## Simple Summary

Crop diseases caused by viruses are a major threat to rice production, especially in Asia. Southern rice black-streaked dwarf virus (SRBSDV) is one such virus, which is spread by the white-backed planthopper (WBPH) but not by the co-occurring brown planthopper (BPH). This study aimed to understand how SRBSDV-infected rice plants affect these two planthoppers differently and how the plant responds. We found that infected plants improved the performance of WBPH by increasing its feeding and lifespan, which may enhance virus spread, although its reproduction was reduced. In contrast, BPH showed lower survival and reproduction on infected plants. We also found that infected plants activated their defense system more strongly when attacked by the WBPH, but not by the BPH. These results show that SRBSDV can change rice plants in ways that favor its transmission by supporting its insect carrier while disadvantaging competing insects. This knowledge can help develop more effective and sustainable strategies for managing rice pests and reducing virus spread.

## 1. Introduction

Plant viruses, with over 1600 species identified to date, often cause great damage to crop quality and yield [1,2]. Approximately 80% of plant viruses depend on insect vectors for transmission, for which piercing-sucking insects, such as planthoppers, aphids, whiteflies, and leafhoppers, are the principal vectors [3,4].

Plants have evolved complex defense mechanisms to cope with diverse biotic and abiotic stresses [5], in which plant hormones play key roles [6,7]. Salicylic acid (SA) is a hormone identified to participate in plant defense against piercing-sucking insects and pathogens including viruses [8,9,10]. White (1979) [11] demonstrated for the first time that exogenous application of SA or aspirin (acetylsalicylic acid) enhanced tobacco resistance to tobacco mosaic virus (TMV). Later studies showed that virus infection induces a marked increase in endogenous SA levels in the majority of plants, accompanied by the activation of pathogenesis-related genes [12,13,14,15,16]. Many phloem sap-feeding insects have also been shown to elicit the SA-signaling pathway [9,17,18,19]. These findings have established the critical role of SA in the plant immune responses to viruses and piercing-sucking herbivores. In plants, SA is biologically synthesized through the shikimic acid pathway with phenylalanine as precursor and, more importantly, through the isochorismate pathway [2,15,20,21]. Interestingly, plants can activate distinct defense pathways depending on the nature of biotic stresses, which can, in turn, influence subsequent plant interactions with other organisms [22,23]. Such plant-mediated interactions play a central role in shaping the arthropod community structure [24,25], especially in the case of sympatric species that occupy the same trophic level and compete for identical resources [25].

Accumulating evidence shows that plant viruses can modulate their transmission efficiency by altering host defense responses, and in some cases by modifying plant nutritional quality and morphological traits [26,27,28,29,30,31]. These virus-induced changes strongly affect the performance and feeding behaviors of vector insects [32,33]. Plant viruses transmitted in a persistent propagative manner typically establish intimate and long-lasting associations with their insect vectors, generating both direct and indirect effects on vector biology [34,35]. Direct effects arise from viral persistence within the vector and can alter survival, fecundity, development, feeding behaviors, and host selection [36,37]. Indirect effects occur when virus-induced physiological and defensive changes in host plants subsequently influence vector population dynamics and other herbivorous insects in the community [38,39,40,41].

Rice is one of the most important staple foods for the world population. The southern rice black-streaked dwarf virus (SRBSDV), a *Fiji* virus transmitted by the white-backed planthopper (WBPH), *Sogatella furcifera*, in a persistent propagative manner [42], has become one of the most destructive rice pathogens in east Asia and southeast Asia, leading to significant yield reduction [43], despite much effort that has been made in recent years to explore environment-friendly ways for the suppression of the viral spread [44,45,46]. The brown planthopper (BPH), *Nilaparvata lugens*, does not transmit SRBSDV, although it occurs sympatrically with WBPH [47] and features the common feeding habits (piercing-sucking phloem sap from leaf sheath). It is interesting to know if SRBSDV infection in rice plants affects the performance of WBPH and BPH equally. There have been reports on the rice plant-mediated indirect interaction between SRBSDV and WBPH or BPH. In one study, SRBSDV indirectly prolongs WBPH female longevity [33]. In another [48], the vector WBPH shows increased fecundity and prolonged nymphal duration while reduced female weight and longevity when feeding on infected plants, and the non-vector BPH shows decreased nymphal survival and shortened longevity. However, the study of Lei et al. [33] focused exclusively on the vector WBPH and did not evaluate whether virus-induced plant responses differentially affect vector and non-vector planthoppers. Although Xu et al. [48] compared the performance of WBPH and BPH, they used naturally infected field plants and did not include mock-inoculated controls, making it difficult to separate virus effects from environmental variation. More importantly, neither study investigated the plant signal responses that may be responsible for the differential performance of vector and non-vector planthoppers. Therefore, the role of SA-mediated defense signaling in shaping these interactions remains unclear.

This study aims to measure the effects of SRBSDV-rice plant interaction on performance of the vector WBPH and the non-vector BPH and to analyze the phytohormonal responses underlying such effects. The performance indicators including nymphal survival and duration and adult fecundity and longevity were measured using 2 × 2 factorial designs combining planthopper species (WBPH vs. BPH) and SRBSDV infection (mock-infected vs. infected). Feeding amount was also determined in adults in this factorial design. Endogenous SA levels and the expression of key genes ICS1 and NPR1 in the SA signaling pathway were analyzed in treatments combining planthopper infestation (uninfested, WBPH infested, BPH infested, and co-infested) and SRBSDV infection (mock-infected vs. infected). These designs test the hypotheses that: (1) SRBSDV-plant interaction will not have an influence on non-vector performance and influence the vector in a way to benefit virus transmission; and (2) the performance patterns echo the patterns of phytohormonal responses.

## 2. Materials and Methods

### 2.1. Insects and Plant

Initial colonies of WBPH and BPH were collected from the Guilin Experimental Station, Ministry of Agriculture and Rural Affairs, Xing’an County, Guangxi, China (25°36′18″ N, 110°42′16″ E), and have been maintained for more than two years on potted rice plants (var. Taichung Native 1, TN1) in separate insect-proof cages. Insect rearing and the following experiments were all performed at 26 ± 2 °C, 70 ± 10% relative humidity (RH), and with a 16L:8D photoperiod.

SRBSDV-infected rice plants were established according to previous reports [31,44]. Seven-day-old rice seedlings (var. Diantun 502) grown in plastic pots with soil substrate in an insect-proof greenhouse were individually transplanted to a cup (9 cm height) containing nutrient soil (peat moss, vermiculite, organic fertilizer and perlite in a 10:10:10:1 ratio by volume). Five viruliferous WBPH 3/4 instar nymphs were collected in a parafilm sachet and attached to the stem of a 15 d old rice plant for virus inoculation. The sachet was removed 5 d later and the plant was maintained until used in the experiments at 30 d post virus inoculation. The plant was designated as the infected plant, whose viral infection status was further confirmed by one-step reverse transcription polymerase chain reaction (RT-PCR) [49], which was also performed to confirm the viruliferous status of the WBPH nymphs. Mock-infected plants were obtained in the same way, except that non-viruliferous WBPH nymphs were used.

### 2.2. Planthopper Performance

**Figure A1:**
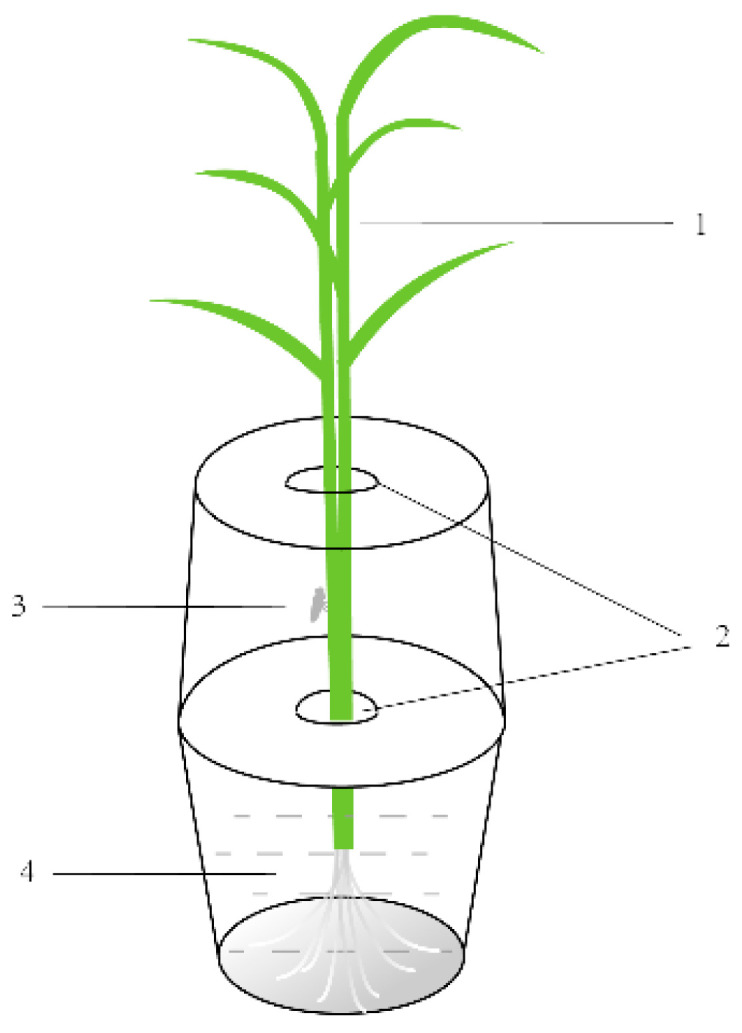
An arena for testing planthopper performance. (1) Rice plants, (2) cotton wool to secure the plant and prevent insects from escaping, (3) planthoppers, and (4) nutrient soil.

An arena was used in the performance tests, which consisted of two cups, a lower cup (9 cm height) filled with nutrient soil and an upper cup put upside down on the lower cup, and a rice stem (either infected or mock-infected) passed through a hole perforated at the centers of the upper cup bottom and of a filter paper lined between the openings of the two cups, with the roots planted in the nutrient soil in the lower cup (Figure A1) [46].

To measure planthopper nymphal duration and survival, a newly hatched nymph of either WBPH or BPH was introduced onto the rice stem in the upper cup of the arena. The nymph was observed twice daily (at 9:00 and 17:00) until adult. For each treatment, 55 nymphs were tested. Nymphal developmental duration (from newly hatched nymph to adult) and survival (the total number of emerged adult planthoppers/the total number of newly hatched nymphs × 100) were calculated.

For planthopper adult longevity and fecundity tests, a pair of newly emerged macropterous adults (<24 h) was introduced into a test cup and observed daily. Nymphs, if there are any in the cup, were recorded and then removed. Upon the death of the female, the leaf sheath was dissected under a stereomicroscope (Guilin Microtech Optical Instrument Co., Ltd, Guilin, China) to record the number of unhatched planthopper eggs therein. The female adult longevity was calculated from the dates of emergence and death. Fecundity was calculated as the sum of nymph numbers and unhatched egg number. For each treatment, 15–20 pairs of adults were tested.

### 2.3. Feeding Amount

**Figure A2:**
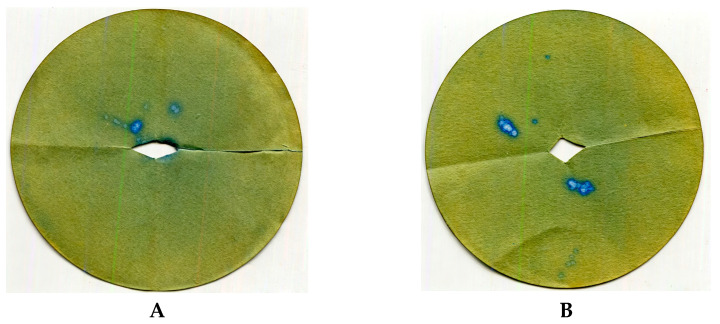

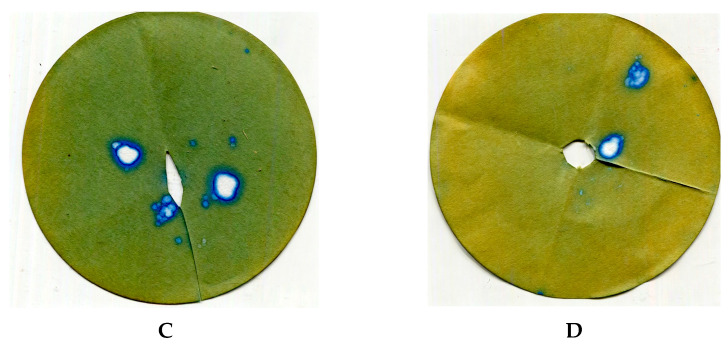
Representative filter paper showing honeydew spots (blue areas) excreted by female planthoppers after 24 h of feeding under different treatments. (**A**) WBPH feeding on mock-infected plant; (**B**) WBPH feeding on SRBSDV-infected plant; (**C**) BPH feeding on mock-infected plant; (**D**) BPH feeding on SRBSDV-infected plant. A larger honeydew area indicates a greater feeding amount.

The planthopper feeding amount was measured in the above arena using the bromocresol green filter paper method, as shown in Figure A2 [46]. A piece of filter paper (6 cm in diameter) soaked in 0.2% bromocresol green ethanol solution was lined between the openings of the two cups. One 2-day-old female macropterous planthopper, starved for 2 h, was introduced onto the rice stem in the upper cup. When the insect fed, it excreted honeydew onto the filter paper, which turned the paper blue. After 24 h, the filter paper was removed, and the area of honeydew spots was measured using a millimeter graph paper. For each treatment, 15 females were tested.

### 2.4. Salicylic Acid Level and Gene Expression

To decipher the plant phytohormonal responses to virus infection and planthopper infestation, phytohormone levels and expression of associated genes were analyzed in eight plant treatments, i.e., mock-infected uninfested plants, infected uninfested plants, infected WBPH infested plants, infected BPH infested plants, infected co-infested plants, mock-infected WBPH infested plants, mock-infected BPH infested plants, and mock-infected co-infested plants. The mock-infected/infected plants were obtained as described above. The infected or mock-infected plants were infested with WBPH or BPH in a glass tube (3 cm × 8 cm) to obtain infested plants. Briefly, a plant transplanted into the tube was infested with 20 3/4 instar nymphs of WBPH or BPH, or with 10 WBPH and 10 BPH nymphs in the case of co-infestation. The leaf sheaths of the infested plants were harvested at 72 h post infestation and flash frozen in liquid nitrogen before they were stored at −80 °C until total RNA extraction or phytohormone analysis. All infected plants used for phytohormone and gene-expression analyses were confirmed as SRBSDV-positive by RT-PCR. The identical procedure followed for uninfested plants, except that the plants were not exposed to planthopper infestation. Four to six biological repetitions were exercised for each treatment, with 3 plants pooled as a repetition.

For the quantification of SA, leaf sheaths were obtained from the frozen rice stems, weighed, and ground in liquid nitrogen to powder before being transferred to a 2 mL microcentrifuge tube. With addition of 1 mL methanol-water-formic acid (15:4:1, *v*/*v*/*v*), the tube was placed in an ultrasonic oscillator (30 W, 40 kHz) for 20 min and then stored in a −20 °C refrigerator for 16 h. After centrifugation at 10,000 rpm for 10 min at 4 °C, the supernatant was collected and the residue in the tube was extracted twice with 500 μL methanol-water-formic acid, and the supernatants were combined. The combined supernatant was dried using a vacuum concentrator (Eppendorf, Hamburg, Germany), reconstituted with 250 μL of methanol-water-acetic acid (90:10:0.05), and then purified with addition of 50 mg C18 adsorbent and 150 mg of anhydrous magnesium sulfate and thorough shake. After centrifugation at 12,000 rpm for 5 min, the supernatant was passed through a 0.22 μm PES filter membrane and stored in a brown sample vial in a −20 °C refrigerator for later analysis. The samples were analyzed using the UPLC-MS/MS ultra-performance liquid chromatography (Waters, Milford, MA, USA), as described by Pan et al. (2010) [50] and Liu et al. (2012) [51].

The expression of SA signaling pathway marker genes *ICS1* and *NPR1* was measured using OsActin and UBQ5 as internal reference genes and the corresponding primer sequences in Table A1. Total RNA was extracted using the TRIzol reagent (Thermo Fisher Scientific, Waltham, MA, USA) following the manufacturer’s instructions. First-strand cDNA was synthesized from 1 μg total RNA using the PrimeScript RT reagent Kit with gDNA Eraser (Takara, Dalian, China) according to the manufacturer’s protocol. Quantitative real-time PCR (qRT-PCR) was performed using Bestar SybrGreen qPCR mastermix (DBI Bioscience, Ludwigshafen, Germany) on an Applied Biosystems 7500 Real-Time PCR System (Thermo Fisher Scientific, USA). The 20 μL PCR reaction contained 10 μL Bestar SybrGreen qPCR mastermix, 0.5 μL forward primer (10 μM), 0.5 μL reverse primer (10 μM), 0.04 μL 50× ROX Reference Dye, 1 μL cDNA template, and 7.96 μL RNase-free ddH_2_O. The thermal cycling program was as follows: initial denaturation at 95 °C for 2 min, followed by 40 cycles of denaturation at 95 °C for 10 s and annealing/extension at 60 °C for 31 s. Relative transcript levels of target genes were calculated using the 2^−ΔΔCt^ method.

### 2.5. Statistical Analysis

The data of fecundity and feeding amount were subjected to two-way analysis of variance (ANOVA) to examine the significant influence of insect species and plant virus infection. Owing to variance heterogeneity, a generalized linear model (GLM) with a Gamma error distribution was used to analyze nymphal duration and adult longevity. The data of plant SA content and the expression of SA related genes were also analyzed using two-way ANOVA for the significant effects of planthopper infestation and plant virus infection. The means were separated by Tukey’s HSD test. All statistical analyses were performed using DPS version 19.05. The proportional data of planthopper nymphal survival were compared between treatments using the Marascuillo procedure [52].

## 3. Results

### 3.1. Planthopper Performance

For planthopper nymphs confined with SRBSDV mock-infected or infected 30 d old rice plants (Table 1), WBPH nymphs showed no difference in survival, whereas BPH nymphs exposed to infected plants survived less than those exposed to mock-infected plants by 18.1% (the Marascuillo procedure). Female nymphal duration was not affected by planthopper species (*F*_1,102_ = 0.006, *p* = 0.939) or plant virus infection (*F*_1,102_ = 2.334, *p* = 0.126) or their interaction (*F*_1,102_ = 0.583, *p* = 0.445). In contrast, male nymphal duration was significantly influenced by plant virus infection (*F*_1,91_ = 4.282, *p* = 0.039) and the interaction between planthopper species and plant virus infection (*F*_1,91_ = 8.273, *p* = 0.004) but not by planthopper species (*F*_1,91_ = 1.784, *p* = 0.182). WBPH male nymphs confined with mock-infected plants lived for 1–2 d shorter than the other three male nymph treatments (Tukey’s HSD test, *p* ≤ 0.016).

**Figure 1:**
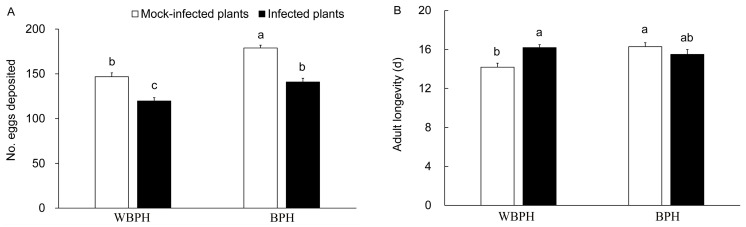
Effects of mock-infected and SRBSDV-infected rice plants on fecundity (**A**) and longevity (**B**) of WBPH and BPH adults. Different letters over the bars indicate significant differences (Tukey’s HSD, *p* < 0.05).

For planthopper adults confined with SRBSDV mock-infected or infected 30 d old rice plants (Figure 1), fecundity differed significantly between planthopper species (*F*_1,62_ = 50.08, *p* < 0.001) and also between virus infection status of rice plants (*F*_1,62_ = 73.82, *p* < 0.001) (Figure 1A). BPH females showed the highest fecundity when confined with the mock-infected plants, and WBPH females deposited the fewest number of eggs when confined with the infected plants. Fecundity of WBPH and BPH was reduced significantly on the infected plants in comparison to the mock-infected plants by 18.5% and 21.1%, respectively (Tukey’s HSD test, *p* < 0.001).

Adult longevity was not significantly affected by planthopper species (*F*_1,125_ = 3.238, *p* = 0.072) or plant virus infection (*F*_1,125_ = 2.337, *p* = 0.126) but by their interaction (*F*_1,125_ = 11.168, *p* = 0.001) (Figure 1B). WBPH adults confined to mock-infected plants lived two days shorter than WBPH to infected plants and BPH to mock-infected plants (Tukey’s HSD test, *p* ≤ 0.006).

The prolonged nymphal development of WBPH on SRBSDV-infected plants extends the period during which nymphs can acquire the virus, while extended adult longevity increases the time available for virus inoculation. Both may enhance overall transmission efficiency. In contrast, the reduced survival and fecundity of the non-vector BPH on infected plants may suppress its population abundance and in turn alleviate interspecific competitive pressure on WBPH.

### 3.2. Feeding Amount

**Figure 2:**
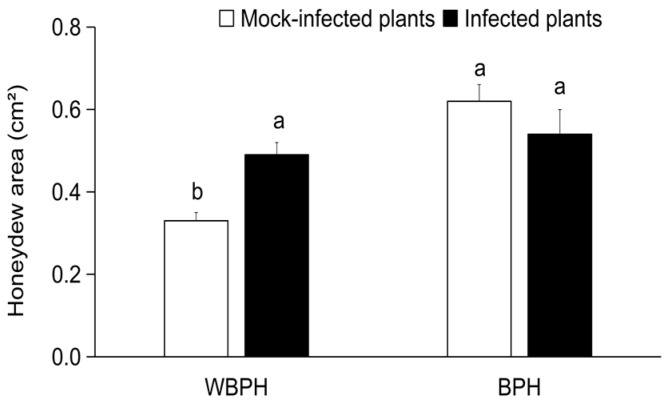
Effects of mock-infected and SRBSDV-infected rice plants on feeding amount of WBPH and BPH adults. Different letters over the bars indicate significant differences (Tukey’s HSD, *p* < 0.05).

Honeydew excretion was measured to represent feeding amount in the planthopper female adults confined with SRBSDV mock-infected or infected 30 d old rice plants. Feeding amount differed significantly between planthopper species (*F*_1,53_ = 16.423, *p* < 0.001) and the magnitude of this difference depended on whether rice plants were infected with SRBSDV (*F*_1,53_ = 8.366, *p* = 0.006) but not by plant virus infection status alone (*F*_1,53_ = 1.042, *p* = 0.312) (Figure 2 and Figure A2). WBPH females confined with mock-infected plants fed fewer than the insects in the other three treatments (Tukey’s HSD test, *p* ≤ 0.038). The increased feeding amount of WBPH on infected plants is likely to enhance both virus acquisition and inoculation efficiency, thereby promoting viral transmission.

### 3.3. Salicylic Acid Level and Gene Expression

**Figure 3:**
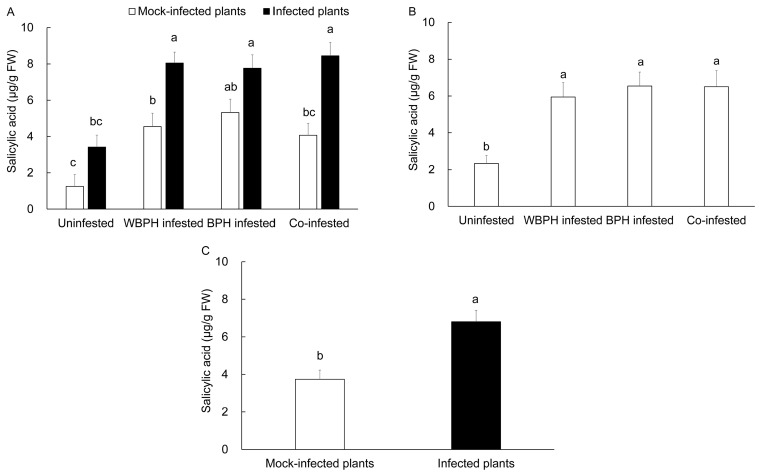
Effects of WBPH and BPH infestation on salicylic acid (SA) levels in rice plants with different virus infection status. (**A**) Interactive effects of planthopper infestation and virus infection on SA levels. (**B**) Effects of planthopper infestation treatments on SA levels. (**C**) Effects of virus infection on SA levels. Different letters over the bars indicate significant differences (Tukey’s HSD, *p* < 0.05).

When the SRBSDV mock-infected vs. infected 30 d old rice plants were exposed planthopper infestation for 72 h, SA level in rice leaf sheaths was significantly influenced by both planthopper infestation (*F*_3,36_ = 17.933, *p* < 0.001; Figure 3B) and virus infection (*F*_1,36_ = 40.243, *p* < 0.001; Figure 3C) but not by their interaction (*F*_3,36_ = 1.072, *p* = 0.376). All the planthopper infested plants except the co-infested mock-infected plants showed a significant increase in SA levels over those in the uninfested mock-infected plants (Tukey HSD test, *p* ≤ 0.019). When the plants were infested by WBPH alone or co-infested, the SA level increased significantly by 43.5% (Tukey’s HSD test, *p* = 0.019) and 51.8% (Tukey’s HSD test, *p* = 0.003) in the infected plants over the mock-infected plants, respectively. In contrast, BPH infestation alone did not trigger a significant difference in SA levels between the mock-infected and infected plants (Tukey’s HSD test, *p* = 0.991) (Figure 3A).

**Figure 4:**
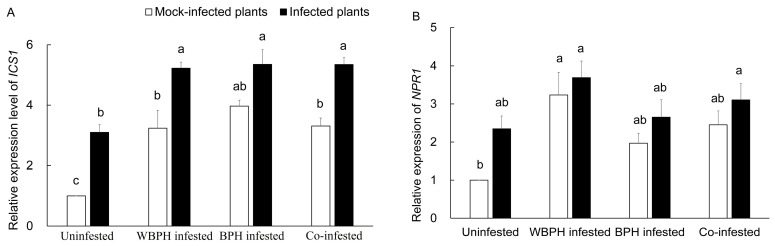
Interactive effects of SRBSDV infection and planthopper infestation on the expression of SA-related genes. (**A**) *ICS1* expression; (**B**) *NPR1* expression. Different letters over the bars indicate significant differences (Tukey’s HSD, *p* < 0.05).

*ICS1* and *NPR1* are two key regulatory genes in the SA-signaling pathway. The expression of *ICS1* was significantly affected by planthopper infestation (*F*_3,31_ = 26.739, *p* < 0.001) and virus infection (*F*_1,31_ = 64.390, *p* < 0.001) but not by their interaction (*F*_3,31_ = 0.485, *p* = 0.6957) (Figure 3A). Likewise, all the planthopper-infested plants showed a significant increase in *ICS1* expression levels over those in the uninfested mock-infected plants (Tukey’s HSD test, *p* ≤ 0.004). When the plants were infested by WBPH alone or co-infested, the *ICS1* expression level increased significantly by 38.1% (Tukey’s HSD test, *p* = 0.005) and 38.1% (Tukey’s HSD test, *p* = 0.004) in the infected plants over the mock-infected plants, respectively. In contrast, BPH infestation alone did not trigger a significant difference in SA levels between the mock-infected and infected plants (Tukey’s HSD test, *p* = 0.102) (Figure 4A).

*NPR1* expression was also significantly influenced by planthopper infestation (*F*_3,31_ = 7.282, *p* = 0.001) and virus infection (*F*_1,31_ = 7.862, *p* = 0.010) but not by their interaction (F_3,31_ = 0.483, *p* = 0.697) (Figure 3B). Significantly higher expression of *NPR1* was seen in the WBPH-infested infected plants (Tukey’s HSD test, *p* = 0.001), WBPH-infested mock-infected plants (Tukey’s HSD test, *p* = 0.011), and co-infested infected plants (Tukey’s HSD test, *p* = 0.018) than in the mock-infected uninfested plants (Figure 4B). The SA levels across all treatments are more closely coupled with the relative expression levels of *ICS1* than with those of *NPR1*.

The finding that SRBSDV-infected plants exhibited stronger SA accumulation and higher expression of SA-related genes in response to WBPH feeding, but not to BPH feeding, indicates that virus infection alters host defense responses in a species-specific manner. Interestingly, despite the enhanced SA response, WBPH showed increased feeding activity and prolonged longevity on infected plants. This suggests that the vector WBPH may possess a greater capacity to tolerate or adapt to SA-mediated defenses than the non-vector BPH. Consequently, the differential activation of SA signaling may contribute to the contrasting performance of vector and non-vector planthoppers on SRBSDV-infected rice plants, although the underlying mechanisms require further investigation.

## 4. Discussion

The interactions between plant phloem-dwelling viruses and herbivorous insects represent exciting frontiers of plant science, which can be either direct or indirect [16]. Direct interaction occurs through virus infection of vectors; indirect interaction may exist between viruses and both vectors and non-vectors through virus infection of host plants. In the current study, we examined the plant-mediated influence of SRBSDV on the performance of the vector WBPH and the non-vector BPH and the related plant phytohormonal responses. WBPH lived longer as male nymphs and adults, fed more but oviposited fewer on the infected plants, which contrasts to reduced nymph survival and fecundity in BPH. SRBSDV infection triggered an increase in salicylic acid (SA) levels and upregulated the expression of SA-related genes (*ICS1* and *NPR1*) in response to WBPH feeding, but not to BPH feeding.

This study revealed that infected rice plants significantly prolonged male nymphal duration and increased female feeding amount and longevity but markedly reduced fecundity of WBPH. Previous studies have revealed that, when feeding on SRBSDV-infected plants, WBPH nymphs live longer [48], whereas WBPH adults show either reduced [48] or prolonged [33] longevity. The specific reasons for the disparity in adult longevity responses to infected plants between different studies are not quite clear; the homogeneity of infected plants used in the tests may have an effect, as they are laboratory-obtained in both the current study and the study by Lei et al. (2014) [33], while they are field-collected in the tests by Xu et al. (2016) [48]. Feeding behaviors are central to viral acquisition and transmission [36,53,54], in that increased feeding favors viral acquisition and/or inoculation. Unlike some persistent viruses that enhance vector fecundity to promote vertical transmission, SRBSDV-infected rice plants significantly suppressed WBPH fecundity in the present study, which differs from the report by Xu et al. (2016) [48]. Similar fecundity-reducing effects have been documented for tomato yellow leaf curl virus (TYLCV) on the vector Bemisia tabaci [50], as well as for rice gall dwarf virus (RGDV) on the vector Recilia dorsalis [55]. Although SRBSDV is not transovarially transmitted, the 18.5% reduction in WBPH fecundity observed on infected plants could potentially reduce the abundance of future vector populations. However, this potential negative effect may be offset by enhancements in other vector traits that are more directly associated with virus transmission. In particular, reduced fecundity may contribute to the prolonged adult longevity observed in WBPH females through the well-documented reproduction–longevity trade-off in short-lived insects [56,57]. In addition, infected plants promoted feeding activity, which may increase the probability of virus acquisition and inoculation, while extended adult longevity enlarges the temporal window available for virus transmission. Taken together, these findings suggest that SRBSDV-infected plants induce a reproduction–transmission trade-off in WBPH, whereby reduced reproductive output is accompanied by enhanced feeding activity and prolonged lifespan. Similar trade-offs have been documented in other virus–vector interactions, where transmission-related traits are favored despite reproduction costs to the vector. Therefore, from the perspective of viral fitness, the cost of reduced vector fecundity is likely compensated for, or even outweighed, by gains in transmission efficiency. In contrast to the vector WBPH, for the non-vector BPH, SRBSDV-infected rice plants reduced nymph survival and fecundity in this study. Xu et al. (2016) [48] observed similar results of reduced BPH nymphal survival but not reduced fecundity. Anyway, the low performance of BPH on infected plants could allow WBPH a competitive edge over BPH in the field populations and may help virus spread. Taken together, the current results conform with the general phenomenon that insect vectors often perform better on virus-infected plants [58].

Plant virus infection modulates the defense responses of host plants, often altering the profiles of defensive secondary metabolites. These virus-induced changes can in turn influence the development and behaviors of vector insects in a way that facilitates virus transmission [59]. One of the key mechanisms involves the SA signaling pathway. For example, TYLCV infection increased SA levels in tomato leaves [60], cucumber mosaic virus (CMV) modified SA metabolism in host plants [61], and tomato spotted wilt virus (TSWV) induced SA-related gene expression [14]. Consistent with these findings, we observed a significant increase in SA levels in rice sheaths of SRBSDV-infected 30 d old plants, supporting the view that virus-induced reprogramming of SA signaling is a common plant response to infection. Plants activate specific defense pathways in response to feeding by different insect species, and such responses often vary in both pathway specificity and intensity [17,62]. Virus infection may further fine-tune host plant defense signaling, thereby reshaping plant–insect interactions. Our results demonstrate differential SA defense responses in the SRBSDV-infected 30 d old rice plants to 72 h planthopper infestation, which were markedly enhanced in WBPH infestation but not in BPH infestation. And the relative expression levels of *ICS1* across all treatments closely follow those of SA levels. Similar results have been reported in a previous study, where SA levels showed a temporal increase pattern in WBPH-infested rice plants, corresponding to the pattern of relative expression levels of SA-marker genes (*ICS1* and *NPR1*) [63]. Despite the SA defense, the vector WBPH performs better than the non-vector BPH on SRBSDV-infected plants. On one hand, virus-activated SA defense may reduce host suitability and impose stronger constraints on non-vector insects, while the vector may tolerate or adapt to the defense environment, a pattern commonly observed in virus–vector systems shaped by long-term coevolution [59]. On the other hand, plant viruses may, through certain ways, suppress plant defenses and promote vector performance. In TYLCV, a C2 protein suppresses plant defenses by interacting with plant ubiquitin [58]. The specific mechanisms for the contrasting performance of WBPH and BPH on SRBSDV-infected plants need to be further explored.

## 5. Conclusions

In summary, SRBSDV infection of rice plants differentially affects the performance of vector and non-vector planthoppers. While WBPH shows enhanced feeding and prolonged longevity on infected plants, the non-vector BPH experiences reduced survival and fecundity. SRBSDV infection triggers an increase in SA levels and upregulates the expression of SA-related genes (*ICS1* and *NPR1*) in response to WBPH feeding, but not to BPH feeding. These interactions of SRBSDV-rice plant-vector and non-vector planthoppers may favor virus transmission while impairing the fitness of a competing non-vector, which adds new findings to previous reports and advances current understanding of virus–plant–herbivore ecology.

## Figures and Tables

**Table 1:** Survival and duration of WBPH and BPH nymphs confined to SRBSDV infected vs. mock-infected 30 d old rice plants. Nymphal survival is calculated from a single sample. Nymph duration (mean ± standard error) is calculated from 55 nymphs. Different letters following means within a column denote significant difference (^1^: Marascuillo procedure; ^2^: Tukey’s HSD test. *p* = 0.05).

Insect	Virus Infection	Nymphal Survival (%) ^1^	Nymphal Duration (d) ^2^	
			Female	Male
WBPH	Mock-infected	96.4 a	13.9 ± 1.7 a	12.6 ± 1.3 b
	Infected	87.3 ab	14.6 ± 1.6 a	14.0 ± 1.7 a
BPH	Mock-infected	94.5 a	14.1 ± 1.1 a	13.8± 1.0 a
	Infected	76.4 b	14.4 ± 1.7 a	13.6 ± 1.6 ab

## Data Availability

The original contributions presented in this study are included in the article. Further inquiries can be directed to the corresponding author.

## Abbreviations

The following abbreviations are used in this manuscript:

WBPH	White-backed planthopper
BPH	Brown planthopper
SRBSDV	Southern rice black-streaked dwarf virus
SA	Salicylic acid

## Appendix A

**Table A1:** The primers of SA signaling pathway marker genes and internal reference genes.

Name	GenBank No.	Sequence (5′–3′)
*ICS1*	AK120689	TATGGTGCTATCCGCTTCGAT CGAGAACCGAGCTCTCTTCAA
*NPR1*	AY923983	TTTCCGATGGAGGCAAGAG GCTGTCATCCGAGCTAAGTGTT
*UBQ5*	AK061988	AACCACTTCGACCGCCACT GTTCGATTTCCTCCTCCTTCC
*OsActin*	AB047313	CAGCACATTCCAGCAGAT GGCTTAGCATTCTTGGGT
